# Screening for Key Pathways Associated with the Development of Osteoporosis by Bioinformatics Analysis

**DOI:** 10.1155/2017/8589347

**Published:** 2017-03-30

**Authors:** Yanqing Liu, Yueqiu Wang, Yanxia Zhang, Zhiyong Liu, Hongfei Xiang, Xianbo Peng, Bohua Chen, Guyou Jia

**Affiliations:** ^1^Department of Geriatric Medicine, Jining No. 1 People's Hospital, Jining, Shandong 272011, China; ^2^Department of Joint Branch, Jining No. 2 People's Hospital, Jining, Shandong 272000, China; ^3^Department of Public Health, Jining Psychiatric Hospital, Jining, Shandong 272000, China; ^4^Department of Prevention and Health, Center for Disease Control and Prevention of Jining City, Jining, Shandong 272000, China; ^5^Department of Spine Surgery, The Affiliated Hospital of Qingdao University, Qingdao, Shandong 266003, China; ^6^Department of Orthopedics, Shandong Provincial Qianfoshan Hospital, Shandong University, Jinan, Shandong 250014, China

## Abstract

*Objectives.* We aimed to find the key pathways associated with the development of osteoporosis.* Methods.* We downloaded expression profile data of GSE35959 and analyzed the differentially expressed genes (DEGs) in 3 comparison groups (old_op versus middle, old_op versus old, and old_op versus senescent). KEGG (Kyoto Encyclopedia of Genes and Genomes) pathway enrichment analyses were carried out. Besides, Venn diagram analysis and gene functional interaction (FI) network analysis were performed.* Results.* Totally 520 DEGs, 966 DEGs, and 709 DEGs were obtained in old_op versus middle, old_op versus old, and old_op versus senescent groups, respectively. Lysosome pathway was the significantly enriched pathways enriched by intersection genes. The pathways enriched by subnetwork modules suggested that mitotic metaphase and anaphase and signaling by Rho GTPases in module 1 had more proteins from module.* Conclusions.* Lysosome pathway, mitotic metaphase and anaphase, and signaling by Rho GTPases may be involved in the development of osteoporosis. Furthermore, Rho GTPases may regulate the balance of bone resorption and bone formation via controlling osteoclast and osteoblast. These 3 pathways may be regarded as the treatment targets for osteoporosis.

## 1. Introduction

Primary osteoporosis is a polygenetic disease characterized by an imbalance of bone homeostasis including microarchitectural deteriorations and low bone mineral density [1]. It is reported that approximately 5.5 million men and 22 million women had osteoporosis in the European Union in 2010 [2]. Risk factors for osteoporosis include gender, advanced age, and diminished sex steroid production after menopause and in elderly individuals and so on [3, 4]. Thus, it is important to get the molecular mechanisms for osteoporosis and then find the effective treatment methods for it.

It has been reported that strontium results in increased bone formation and decreased bone resorption by the modulation of several pathways including CaSR, ERK1/2-MAPK, and NFATc/Wnt signaling pathways [5]. One study showed that RANKL (receptor activator of NF-*κ*B ligand)/RANK (receptor activator of NF-*κ*B)/OPG (osteoprotegerin) signaling system was essential for skeletal homeostasis, and disruption of it led to inhibition of bone resorption in vitro [6]. The bone formation inhibitor sclerostin encoded by SOST binds in vitro to low density LRP5/6 (lipoprotein receptor-related protein) Wnt coreceptors, thereby inhibiting Wnt/*β*-catenin signaling, a central pathway of skeletal homeostasis, and LRP5 deficiency results in OPPG (osteoporosis-pseudoglioma), whereas SOST deficiency induces lifelong bone gain in mice and humans [7]. Azuma et al. indicated that the SXR/PXR (Nuclear Receptor Subfamily 1, Group I, Member 2) dependent signaling pathway could mediate the protective effects of vitamin K for bone [8]. Pineda et al. suggested that antioxidant pathways played important roles in bone homeostasis [9]. Furthermore, functional polymorphisms of the* P2X7* (Purinergic Receptor P2X, Ligand Gated Ion Channel, 7) receptor gene are related to osteoporosis [10]. Two single nucleotide polymorphisms of rs4237 and rs2501431 in* CNR2* (Cannabinoid Receptor 2) gene may result in postmenopausal osteoporosis in Han Chinese women [11].* Mettl21c* (methyltransferase-like 21C) may play bone-muscle pleiotropic roles through the regulation of the NF-*κ*B signaling pathway [12]. However, the underlying mechanisms or key regulating factors for osteoporosis are not fully understood.

Bone marrow mesenchymal cells (BMSCs) are the major source of osteoprogenitor cells resulting in remodeling of bone in adults [13]. The former researches using the data of GSE35959 demonstrated that nuclear factor I-C (NFI-C) regulated osteoblast differentiation [14], mechanical stimulation affected genes expression associated with osteogenic differentiation of BMSCs through the regulation of HDAC1 (Histone Deacetylase 1) [15], or the transcriptional profile of MSC populations in primary osteoporosis showed overexpression of osteogenic inhibitors [16]. In contrast to previous studies, we downloaded this data and analyzed the differentially expressed genes (DEGs) in 3 comparison groups. KEGG (Kyoto Encyclopedia of Genes and Genomes) pathway enrichment analyses were carried out. Besides, Venn diagram analysis and gene functional interaction (FI) network analysis were performed. We aimed to understand the key pathways associated with the development of osteoporosis and then find the effective treatment methods for it.

## 2. Materials and Methods

### 2.1. Expression Profile Data

The expression profile data of GSE35959 deposited by Benisch et al. was downloaded from the GEO (Gene Expression Omnibus, https://www.ncbi.nlm.nih.gov/geo/) database [16]. A total of 5 middle aged human mesenchymal stem cells (MSC) samples (middle), 4 old elderly MSC samples (old), 5 primary osteoporosis elderly MSC samples (old_op), and 5 senescent MSC samples (senescent) were included in this study. The data were based on the platform of GPL570 (Affymetrix Human Genome U133 Plus 2.0 Array, Affymetrix, Inc., Santa Clara, California, USA).

### 2.2. Data Preprocessing

The raw data were preprocessed by R package affy (version: 3.24.15) [17] in Bioconductor (http://www.bioconductor.org/). Background correction, normalization between arrays, and calculated expression were included in the process of preprocessing. The probe ID was transformed into gene symbol combined with annotation files of the platform.

### 2.3. DEGs Analysis

Significant *p* value for DEGs in primary osteoporosis elderly MSC versus middle aged MSC (old_op versus middle), primary osteoporosis elderly MSC versus old elderly MSC (old_op versus old), and primary osteoporosis elderly MSC versus senescent MSC (old_op versus senescent) were analyzed with *t*-test in limma (version: 3.24.15) [18]. The *p* value was adjusted as FDR (false discovery rate) values by BH (Benjamini-Hochberg) [19]. FDR < 0.05 and |log2FC| ≥ 1 were used as cut-off criterion for DEGs.

### 2.4. KEGG (Kyoto Encyclopedia of Genes and Genomes) Pathway Enrichment Analysis

KEGG is a database used for putting associated genes into the corresponding pathways [20]. R package clusterProfiler (version: 2.2.7) [21] based on KEGG.db annotation package was used to the pathway enrichment analysis. Significant *p* values for enriched DEGs were calculated by hypergeometric distribution, and *p* < 0.05 was set as significantly enriched pathway.

### 2.5. Venn Diagram Analysis for DEGs

VennPlex (http://www.irp.nia.nih.gov/bioinformatics/vennplex.html) [22] can be used to analyze Venn diagram for multiple dataset by using gene expression values and screen out the significant genes. Furthermore, it can display the number of intersection genes that are upregulated, downregulated, and contraregulated, respectively. By using a large set of functional association data including protein and genetic interactions, pathways, coexpression, colocalization, and protein domain similarity, GeneMANIA [23] can find other genes associated with a set of input genes.

DEGs and log2FC of 3 comparison groups were input into VennPlex, and the similarities and differences in 3 comparison groups were observed. Furthermore, KEGG pathways enriched by intersection DEGs in 3 groups were obtained. The correlations among intersection DEGs were analyzed by Cytoscape (version: 3.2.1) [24] app-GeneMANIA.

### 2.6. Gene Functional Interaction (FI) Network Analysis

The ReactomeFIViz app [25] can construct FI network, calculate correlations (average Pearson correlation coefficient) among genes, use the calculated correlations as weights for edges, apply Monte Carlo localization graph clustering algorithm to the weighted FI network, and generate a subnetwork for a list of selected network modules.

Gene functional interaction networks were analyzed with Cytoscape app-ReactomeFIViz. The input dataset was expression matrix of all DEGs, and pathway enrichment analysis for every function module was performed to find biological pathway involved by every module genes (FDR < 0.05). Other ReactomeFI parameters were set as defaults.

## 3. Results

### 3.1. DEGs Analysis

A total of 520 DEGs, 966 DEGs, and 709 DEGs were obtained in old_op versus middle, old_op versus old, and old_op versus senescent groups, respectively (Table 1).

### 3.2. Functional Enrichment Analysis

**Figure 1:**
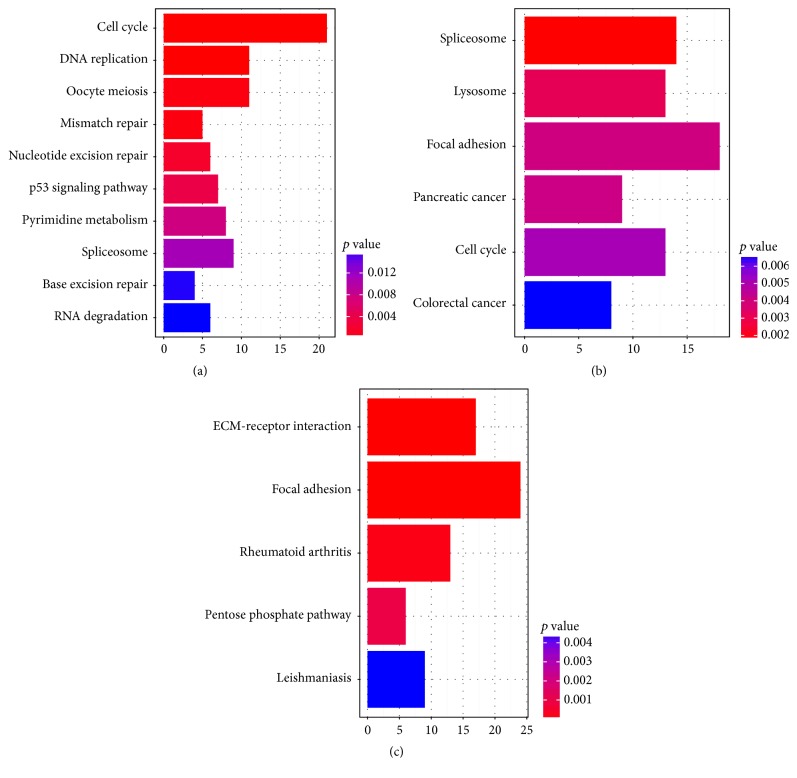
The significantly enriched KEGG (Kyoto Encyclopedia of Genes and Genomes) pathways for 3 comparison groups. (a) Primary osteoporosis elderly MSC versus middle aged MSC; (b) primary osteoporosis elderly MSC versus old elderly MSC; (c) primary osteoporosis elderly MSC versus senescent MSC.

The significantly enriched KEGG pathways for 3 comparison groups were shown in Figure 1. Cell cycle pathway was the significantly enriched pathway in old_op versus middle, and focal adhesion pathway was the significantly enriched pathway in old_op versus old, as well as old_op versus senescent.

### 3.3. Venn Diagram Analysis for DEGs

**Figure 2:**
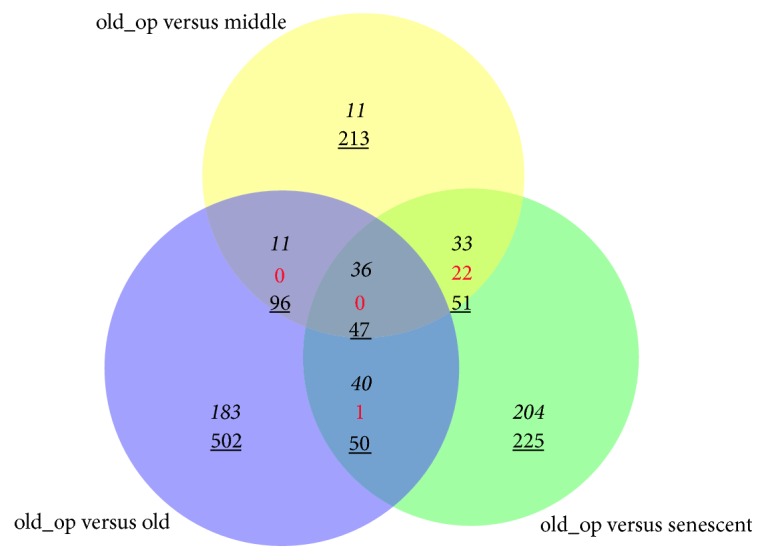
Venn diagram analysis for differentially expressed genes (DEGs).

**Figure 3:**
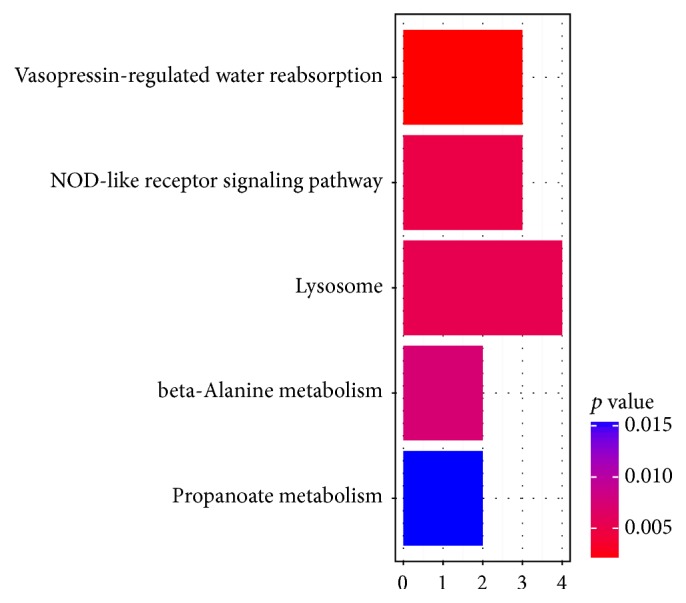
KEGG (Kyoto Encyclopedia of Genes and Genomes) pathways significantly enriched by intersection genes.

**Figure 4:**
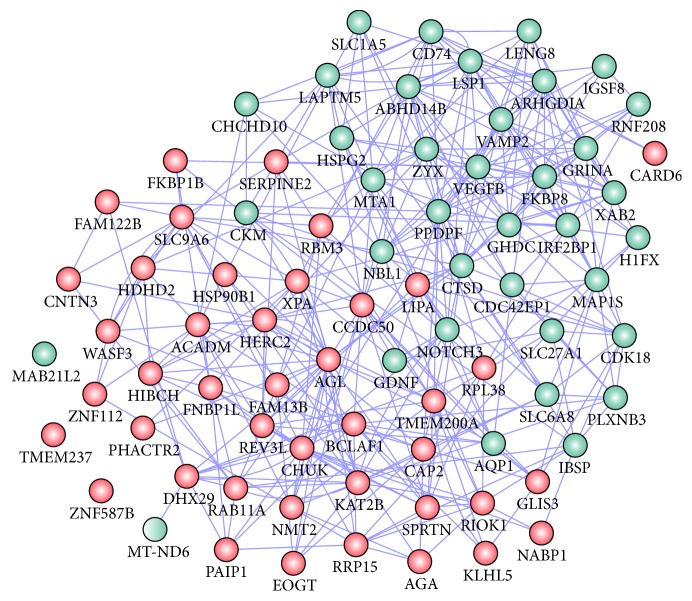
The interaction network among intersection genes. Red nodes: downregulated; green nodes: upregulated.

Venn diagram analysis for DEGs was shown in Figure 2. A total of 36 upregulated genes and 47 downregulated genes were included in DEGs of 3 comparison groups. KEGG pathways significantly enriched by intersection genes were shown in Figure 3. Lysosome pathway enriched by LAPTM5 (Lysosomal Protein Transmembrane 5, upregulated), CTSD (Cathepsin D, upregulated), LIPA (Lipase A, Lysosomal Acid Type, downregulated), and AGA (Aspartylglucosaminidase, downregulated) was the significantly enriched pathways enriched by intersection genes. The interaction network among intersection genes obtained by GeneMANIA analysis was shown in Figure 4, and 79 intersection genes and 367 interaction pairs were included in the interaction network. In addition, this interaction network included 5 association data items (262 coexpressions, 7 physical interactions, 80 genetic interactions, 17 colocalization, and 1 pathway).

### 3.4. Gene Functional Interaction Network Analysis

**Figure 5:**
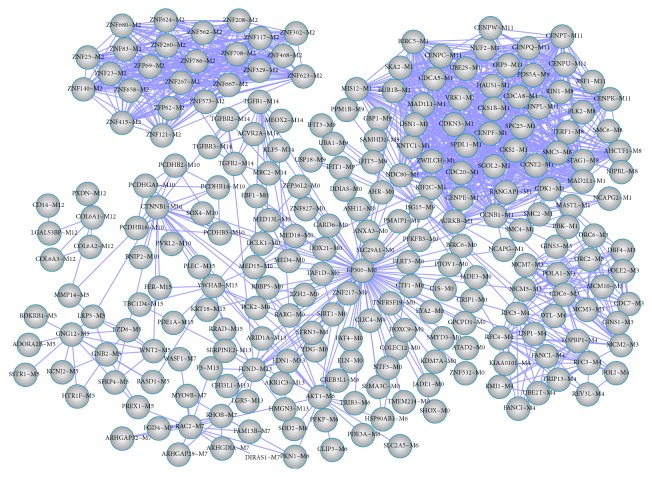
The functional interaction network for differentially expressed genes (DEGs); “gene symbol~M0–15” represents the name of nodes; suffix “M0–15” represents the subnetwork modules that this gene belongs to.

The functional interaction network for DEGs obtained by ReactomeFI was shown in Figure 5, and 240 nodes and 1309 interaction pairs were included in it. Furthermore, this network included 16 significant subnetwork modules (Table 2). Module 0 and module 1 had more nodes. The absolute value of average correlation between subnetwork module genes was high. In addition, pathways enriched by subnetwork modules were shown in Table 3. Mitotic metaphase and anaphase and signaling by Rho GTPases in module 1 had more proteins from module.

## 4. Discussions

In the current study, with the expression profile data of GSE35959, totally 520 DEGs, 966 DEGs, and 709 DEGs were obtained in old_op versus middle, old_op versus old, and old_op versus senescent groups, respectively. Lysosome pathway was the significantly enriched pathways enriched by intersection genes. Furthermore, the pathways enriched by subnetwork modules suggested that mitotic metaphase and anaphase and signaling by Rho GTPases in module 1 had more proteins from module.

Lysosome pathway was the significantly enriched pathways enriched by intersection genes in our present study. RANKL/RANK/OPG signaling system was essential for skeletal homeostasis, and one study showed that RANKL was found to be localized to secretory lysosomes in osteoblastic cells [26]. Yoneshima et al. suggested that lysosomal biogenesis mediated by TFEB (Transcription Factor EB) might be involved in osteoblast differentiation [27]. Furthermore, osteoporosis is characterized by an imbalance of bone resorption and bone formation [28]. Thus, our results are according to the previous studies and show that lysosome pathway plays important parts in the development of osteoporosis.

Furthermore, mitotic metaphase and anaphase in module 1 had more proteins from module in this study. The transcription of* Runx2* mRNA is dependent on mitosis and the translation of it after mitosis in early osteoprogenitors to control the gene expression required for reinforcement of cell fate decisions in committed preosteoblasts [29]. One study indicated that LRP1 (low density lipoprotein receptor-related protein 1) could activate the p42/44 MAPK (mitogen-activated protein kinase) pathway and then lead to the mitosis of osteoblasts [30]. Thus, mitosis is significant for the osteoblasts. Besides, osteoblasts play key roles in the bone formation, and osteoporosis is characterized by the imbalance of bone resorption and bone formation. Therefore, combined with our results, we speculate that mitotic metaphase and anaphase may play key roles in the progression of osteoporosis.

In addition, our study also showed that signaling by Rho GTPases in module 1 had more proteins from module. Rho GTPases may control podosome arrangements in osteoclasts [31]. Brazier et al. showed that the Rho GTPase* Wrch1* (Ras Homolog Family Member U) regulated precursor adhesion and migration of osteoclast [32]. Touaitahuata et al. suggested that Rho GTPases could modulate osteoclast differentiation and bone resorption [33]. Besides, Wan et al. indicated that Rho GTPases controlled TCF/LEF (hepatocyte nuclear factor 4, alpha) activity and nuclear localization of *β*-catenin in osteoblasts under flow [34]. Thus, Rho GTPases may play roles in osteoclast and osteoblast. Rho GTPases may regulate the balance of bone resorption and bone formation via controlling osteoclast and osteoblast. Combined with our results, we think that signaling by Rho GTPases may be involved in the development of osteoporosis.

However, there are several limitations in this study. First, only 19 samples including 5 middle aged MSCs, 4 old elderly MSCs, 5 primary osteoporosis elderly MSCs, and 5 senescent MSCs were included in this study. Second, in Results, the interaction network among intersection genes includes 79 intersection genes and 367 interaction pairs, but there is no significant difference among the weight of these intersection genes. Third, our study is concluded from the bioinformatics analysis of the expression profile data downloaded from the GEO database, and further experiments are needed to verify our results.

## 5. Conclusions

In conclusion, lysosome pathway, mitotic metaphase and anaphase, and signaling by Rho GTPases may be involved in the development of osteoporosis. Furthermore, Rho GTPases may regulate the balance of bone resorption and bone formation via controlling osteoclast and osteoblast. Lysosome pathway, mitotic metaphase and anaphase, and signaling by Rho GTPases may be regarded as the treatment targets for osteoporosis. However, further studies with large samples and verification experiments are needed.

## Figures and Tables

**Table 1:** The number of differentially expressed genes (DEGs) for 3 comparison groups.

Group	Upregulated gene count	Downregulated gene count	Total
old_op versus middle	91	429	520
old_op versus old	270	696	966
old_op versus senescent	336	373	709

**Table 2:** The information of 16 significant subnetwork modules.

Module	Nodes in module	Average correlation
0	51	0.5639
1	37	0.7701
2	24	0.6355
3	14	0.848
4	14	0.7763
5	14	0.5407
6	10	0.5594
7	10	0.4676
8	9	0.7657
9	9	0.7226
10	9	0.5833
11	9	0.5608
12	9	0.5231
13	9	0.3758
14	8	0.3205
15	7	0.546

**Table 3:** Pathways enriched by subnetwork modules.

Module	Gene set	Protein from module	FDR
0	Transcriptional regulation of white adipocyte differentiation (R)	6	2.72*E* − 05
0	Chromatin modifying enzymes (R)	6	2.03*E* − 03
1	Mitotic metaphase and anaphase (R)	23	2.00*E* − 15
1	Signaling by Rho GTPases (R)	21	2.00*E* − 15
3	Synthesis of DNA (R)	11	4.44*E* − 16
3	S phase (R)	11	4.44*E* − 16
4	DNA damage bypass (R)	8	9.99*E* − 16
4	Fanconi anemia pathway (N)	9	9.99*E* − 16
5	Heterotrimeric G-protein signaling pathway-Gq alpha and Go alpha mediated pathway (P)	5	1.74*E* − 05
5	Wnt signaling pathway (P)	6	2.86*E* − 05
6	Protein kinase a at the centrosome (B)	2	6.92*E* − 03
6	Prostate cancer (K)	3	6.92*E* − 03
7	Signaling by Rho GTPases (R)	8	4.73*E* − 10
7	Regulation of RhoA activity (N)	2	1.07*E* − 02
8	Mitotic telophase/cytokinesis (R)	4	7.08*E* − 09
8	Mitotic prometaphase (R)	4	8.56*E* − 06
9	Interferon alpha/beta signaling (R)	5	6.83*E* − 09
9	ISG15 antiviral mechanism (R)	4	6.35*E* − 07
10	Cadherin signaling pathway (P)	6	4.47*E* − 09
10	Wnt signaling pathway (P)	6	7.94*E* − 07
11	Nucleosome assembly (R)	9	5.55*E* − 16
11	Mitotic prometaphase (R)	6	1.36*E* − 10
12	Beta1 integrin cell surface interactions (N)	4	4.71*E* − 08
12	ECM-receptor interaction (K)	3	2.45*E* − 05
13	Formation of fibrin clot (clotting cascade) (R)	2	1.36*E* − 02
13	AP-1 transcription factor network (N)	2	2.12*E* − 02
14	TGF-beta signaling pathway (P)	4	4.87*E* − 06
14	TGF-beta signaling pathway (K)	4	4.87*E* − 06
15	Insulin-mediated glucose transport (N)	2	9.27*E* − 03
15	Class I PI3K signaling events mediated by Akt (N)	2	9.27*E* − 03

The source of pathway database: C: CellMap, R: Reactome, K: KEGG, N: NCI PID, P: Panther, and B: BioCarta.

## Additional Points

*Highlights.* (1) Lysosome pathway may be involved in the development of osteoporosis. (2) Mitotic metaphase and anaphase may play key roles in osteoporosis. (3) Signaling by Rho GTPases may be important for the development of osteoporosis. (4) These 3 pathways may be regarded as the treatment targets for osteoporosis.
